# Effectiveness of Fecal Microbiota Transplantation Treatment in Patients With Recurrent Clostridium difficile Infection, Ulcerative Colitis, and Crohn’s Disease: A Systematic Review

**DOI:** 10.7759/cureus.42120

**Published:** 2023-07-19

**Authors:** Arturo P Jaramillo, Babatope L Awosusi, Javaria Ayyub, Karan Nareshbhai Dabhi, Namra V Gohil, Nida Tanveer, Sally Hussein, Shravya Pingili, Vijaya Krishna Makkena

**Affiliations:** 1 General Practice, California Institute of Behavioral Neurosciences & Psychology, California, USA; 2 Pathology and Laboratory Medicine, California Institute of Behavioral Neurosciences & Psychology, Fairfield, USA; 3 Internal Medicine, California Institute of Behavioral Neurosciences & Psychology, Fairfield, USA; 4 Internal Medicine, Baroda Medical College, Vadodara, IND; 5 Internal Medicine, Allied Hospital/Faisalabad Medical University, Faisalabad, PAK; 6 Internal Medicine, Kakatiya Medical College, Hyderabad, IND; 7 Internal Medicine, Osmania Medical College, Hyderabad, IND

**Keywords:** irritable bowel disease, chron’s disease, ulcerative colitis (uc), fecal microbiota transplantation in clostridium difficile infection, clostridium difficile infection treatment

## Abstract

Cronh’s disease and ulcerative colitis (UC) are diseases with unknown etiologies that cause ongoing inflammation in the gastrointestinal system. Chron's disease causes immunological dysregulation, and UC causes intestinal harm due to immune reactions. According to our study, fecal microbiota transplantation (FMT) has many benefits in the treatment of inflammatory bowel disease (IBD) by restoring intestinal homeostasis and reducing clinical symptoms. In mildly symptomatic patients with UC, an FMT treatment combined with an anti-inflammatory diet can produce remission, which would then be followed by a diet that maintained the anti-inflammatory effects. The efficacy of FMT consists of preventing flares or the consequences of IBD. As a result, we must emphasize that more investigation should be done before developing a therapeutic procedure for FMT in IBD and its associated consequences.

## Introduction and background

The normal gut flora has a significant role in human well-being, and wide imbalances have been linked to certain illnesses [1-2]. The normal gut flora is highly important for human health, and many different types of aberrations are associated with the development of various illnesses [1-2]. The techniques and technology for fecal microbiota transplantation (FMT) are evolving. Some of these studies used treatments, such as donor FMT through enteral tubing, microbiota that are washed before being given, and refined fecal spores [3-4]. FMT is a time-consuming therapy that heals a specific ailment by injecting feces from donors into a damaged gastrointestinal (GI) tract. FMT can be used for a wide variety of GI-related illnesses, such as non-GI and GI problems, including recurrent *Clostridium difficile* infection (CDI), idiopathic constipation, irritable bowel syndrome, and inflammatory bowel disease (IBD) [5-6]. Accordingly, we examined the existing information on how important FMT is in treating cases of recurring infections caused by CDI and IBD.

## Review

Methodology

**Figure 1:**
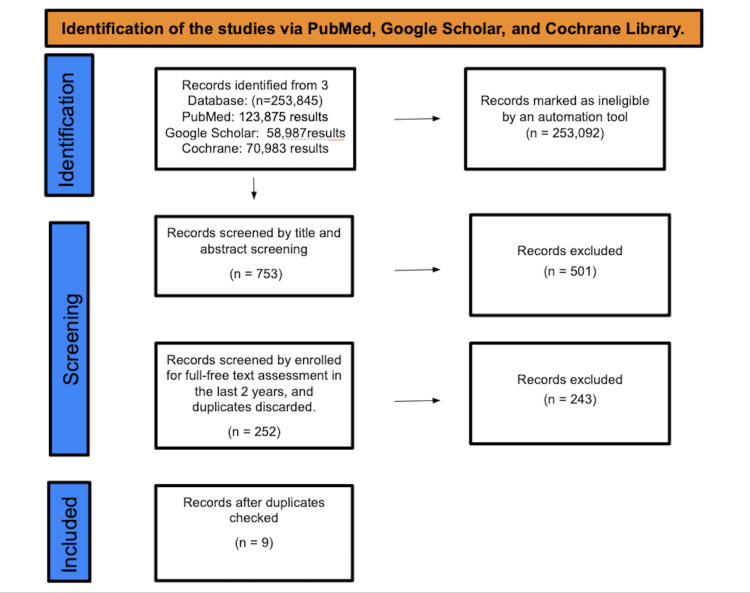
Identification of studies via databases and registers

We used a checklist to record our methods and results after conducting a systematic evaluation of publicly accessible full-length papers. We followed the Preferred Reporting Items for Systematic Reviews and Meta-Analyses (PRISMA) 2020 suggestions, as shown in Figure 1.

Study Duration

This review was started on April 15, 2023 and ended on May 23, 2023.

Search Strategy

We used databases from PubMed, Google Scholar, and Cochraine using the following Medical Subject Headings (MeSH) keywords: "ulcerative colitis," "*Pseudomembranous colitis*," "fecal microbiota transplantation," "inflammatory bowel disease," and "*Clostridium difficile *infection." This study contains clinical trials, systematic review literature, and traditional reviews. We used keywords to find articles corresponding with MeSH terms in PubMed or in Cochrane: "ulcerative colitis," "fecal microbiota transplant," "inflammatory bowel disease," and "*Clostridium difficile* infection." The building block technique was utilized to combine these search phrases, and Boolean operators, such as "OR" and "AND," were employed within the right keywords. Full texts, publicly accessible papers, human trials, and the English language were among the inclusion criteria.

Study Selection and Eligibility

Each article's title and abstract were examined separately by two researchers in order to verify eligibility.

Inclusion and exclusion criteria: We selected the latest literature review, randomized clinical trials (RCTs), and systematic review articles published in the past two years, including papers written in the English language, or if the full-text English-language translation is available. We only included research papers involving human participants.

Articles were excluded if the full text of the papers could not be retrieved. Articles focussing on lifestyle modification and physical activity in obese, prediabetic, and type 2 diabetes mellitus (T2DM) patients. Gray literature and proposal papers were also not included

Data Management

Two separate writers vetted the articles based on their titles and abstracts. Then, suitable abstracts were chosen for full-text examination. The listed studies were evaluated, and if there was any dispute, a third author evaluated that research. The information from the included articles was then extracted: name of the first author, kind of research, year of publication, sample size, and results. In the end, duplicates were also deleted.

Quality Assessment

Different tools were used, such as clinical trials and Cochrane risk of bias assessment tool. The Newcastle-Ottawa questionnaire was used for observational investigations, the Scale for the Assessment of Narrative Review Articles (SANRA) for traditional reviews, and the Assessment of Multiple Systematic Reviews (AMSTAR) tool for systematic reviews and meta-analyses.

Results

A total of 253,845 studies were found after searching in PubMed, Google Scholar, and Cochrane Library. A total of 253,092 were marked as ineligible by an automation tool. A total of 753 studies underwent title and abstract screening, and 501 papers were discarded. The remaining 252 papers were chosen for full-free text evaluation in the previous two years, and after discarding duplicates, resulting in the elimination of 243 studies, nine studies were enlisted for the final collection of data. 

Table 1 shows an in-depth description of the articles we decided to use.

**Table 1:** Data extraction in the included studies UCEIS: Ulcerative Colitis Endoscopic Index of Severity; RCT: randomized control trial; FT: fecal transplantation; SMT: standard medical therapy; FMT: fecal microbiota transplants; CDI: *Clostridium difficile*; FMT/WMT: fecal microbiota transplantation/washed microbiota transplantation; UC: ulcerative colitis; FMFT: fecal microbiota fecal transplantation; UCED: ulcerative colitis exclusion diet; FMT-AID: fecal microbiota transplants and anti-inflammatory diet; N/A: not applicable; SANRA: Scale for the Assessment of Narrative Review Articles; AMSTAR: Assessment of Multiple Systematic Reviews

Author	Year of publication	Study design	Quality appraisal tool	Primary studies	Outcome measure
Tkach et al. [5]	2023	Traditional review	SANRA checklist	712 pilot case report studies, cohort studies, and RCTs were cited and discussed.	FMT was initially utilized successfully in managing individuals with persistent CDI-associated colitis.
Wei et al. [6]	2022	RCT	Cochrane risk of bias assessment tool	64 individuals were identified as having recurrent CDI, as opposed to non-responders and patients who had durable remission after using FMT.	It was discovered that the variations in the microbiota of the gut among results following single therapy with antibiotics, such as fidaxomicin and vancomycin, were not similar following treatment with FMT, demonstrating the different influence of FMT treatment from antibiotics.
Wang et al. [7]	2022	Systematic review	AMSTAR checklist	782 studies were included.	FMT has been used to treat 85 developing disorders. Over the last decade, the quantity of research works on FMT and WMT has expanded considerably.
Boicean et al. [1]	2022	Systematic review	AMSTAR checklist	74 FMT publications used in IBD were selected.	The statistical value of the research conducted so far is not outstanding, and outcome rates remain modest. It is a simple fact that not all of the specifics of the approach's limitations are known.
Stallmach et al. [4]	2022	RCT	Cochrane risk of bias assessment tool	174 random patients with mild to moderately active UC received oral, frozen samples containing multi-donor FMT or FMFT.	The result will be clinical recovery days after the initial FMT or FMFT transfer. The secondary outcomes will include steroid-free therapeutic remission (a Mayo score of 0-1).
Sarbagili et al. [8]	2022	RCT	Cochrane risk of bias assessment tool	Diets were used for patients and donors. RTC studies about adults with active UC were selected.	UCED seems to yield a greater intestinal remission vs. donor FT. A safety assessment commission halted the trial due to its futility.
Poole et al. [3]	2022	Systematic review	AMSTAR checklist	185 studies were collected. The tutor supervised the information recall.	Reliable FMT and its efficacy as a therapeutic choice for a range of disorders, including CDI, delayed transit constipation, inflammatory bowel disease, obesity, and graft-versus-host disease.
Kedia et al. [9]	2022	RCT	Cochrane risk of bias assessment tool	Patients with mild UCEIS on routine medications in an FMT-AID versus optimized SMT.	FMT donors and those with an anti-inflammatory diet efficiently elicited profound recovery in active UC, which was maintained for a year using an anti-inflammatory diet.
Ajay et al. [2]	2022	Traditional review	SANRA checklist	N/A	This study will inform the health community on how these outcomes should be managed throughout a patient's lifetime until new studies of this novel medicine are conducted.

Discussion

Efficacy of FMT in IBD

FMT has been shown to provide substantial advantages over placebo in a sample of people with UC. Its efficacy, however, is contingent on the donor sample administration strategy and the donor sample used. According a study done by Tkach et al., there are now three randomized clinical trials (RCT) evaluating FMT in a population with UC that have shown positive results [5]. The first trial, undertaken by Moayyedi et al., included patients with chronic UC in an RCT composed of a control group and an experimental group. The outcomes showed a decrease in the Mayo score in one quarter of the study population and a minority in those with placebo [10]. The second experiment, carried out by Paramsothy et al., explored how effective FMT was by conducting colonoscopies (CF) on individuals with non-aggressive UC, where 27% of people on FMT and 8% on placebo achieved good results [10]. The last study done by Costello et al. showed how successful FMT was in persons with non-aggressive UC, where it gave outcomes comparable to the last study: remission was seen in 32% of people who were studied receiving fecal transplant, compared with those that received placebo [11].

A systematic review of 25 studies was conducted. The results show that 42% of the over 200 participants with UC who participated in the trial had remission, and more than the half of the study population achieved clinical response. Other cohort studies aimed a remission of 41%, whereas its outcome was 66% [12]. In an established research by Borodi et al., 67.7% of UC patients obtained full CR following FMT and 24.2% achieved partial responses [13].

Effectiveness of FMT in CDI

In a publication by Duarte-Chavez et al., the majority of patients (21/35) had ≥three CDI recurrences before undergoing FMT. Overall, in a total of 35 patients, remission was observed in 86% of patients after FMT treatment [14]. In another study, 16 of 19 patients with diarrhea caused by CDI got better with FMT and an anti-inflammatory diet during the active infection, and none of them got diarrhea again [15]. A randomized control experiment by Youngster et al. showed that the participants' symptoms were alleviated in 70% after one FMT and in 90% of patients after several FMTs [15].

A case report by Schwan et al. describes the first treatment of FMT performed on an individual, who injected FMT during her episodes of recurrent diarrhea and abdominal pain, which led to a beneficial outcome after the first treatment. With that, FMT started to be studied more [16]. Van Nood et al. discovered that vancomycin was less effective in the treatment of recurrent CDI compared to donor feces infusion, which was much more effective [17]. In studies with multiple case reports, FMT was used to treat young people (age between 19 and 28), older people (>65 years old), and pregnant women, and the rectal infusion of homologous feces led to good remission rates. Recent European and Australian research has revealed that antibiotics are unsuccessful, which puts the benefits of FMT treatments as a first-line therapy [11]. A study done retrospectively showed a remission rate of 91.3% in patients with CDI after being treated with FMT in solid organ transplantation [18]. A study reported by Elopre et al. recorded two cases where FMT was use to treat recurrent CDI in HIV-infected people [19].

The FMTv (vancomycin) mechanism of action on gut bacteria was significantly different because those who used vancomycin and fidaxomicin did not differ in terms of responders and nonresponders after their use. Vancomycin and fidaxomicin failed to result in the same improvement in the gut microbiota as FMTv in the responders, although vancomycin resulted in a delayed and minor increase in richness after two months and seven months, respectively. In conclusion, inhibiting the early growth of *C. difficile* is treated by fidaxomicin and vancomycin, but a more complex and presumably more permanent therapeutic effect is given by FMT [20-21].

## Conclusions

FMT is still a viable option for IBD patients. Even though the investigations have not been broad enough to result in the creation of a worldwide protocol, therapeutic results in certain situations have been reported and documented, which motivates more research in this field. Although many more controlled studies are required, the huge rise in data has shown FMT's potential future for numerous gut disorders or disorders outside the stomach. Furthermore, despite improvements in gut microbiota study results, only a few trials have been conducted to learn more about the efficacy of therapeutic approaches that may genuinely affect the microbial community. FMT was first used effectively in modern healthcare to treat recurrent CDI, which is so far the only legally permitted treatment. Nonetheless, the efficacy of FMT is now being explored and enhanced for the better management of a variety of digestive and non-digestive illnesses.
